# Imaging of ventilation and lung injury with low‐frequency tomographic ultrasound

**DOI:** 10.1002/mp.17421

**Published:** 2024-09-23

**Authors:** Andre Vieira Pigatto, Sergio Furuie, Diego Cardénas, Marlis L. Rezende, Raul Lima, Jennifer L. Mueller

**Affiliations:** ^1^ School of Biomedical Engineering Colorado State University Fort Collins Colorado USA; ^2^ Polytechnic School of the University of São Paulo São Paulo Sao Paulo Brazil; ^3^ Department of Clinical Sciences and College of Veterinary Medicine and Biomedical Sciences Colorado State University Fort Collins Colorado USA; ^4^ Department of Mathematics and School of Biomedical Engineering and the Department of Electrical and Computer Engineering Colorado State University Fort Collins Colorado USA

**Keywords:** low‐frequency ultrasound, pulmonary imaging, ultrasound computed tomography

## Abstract

**Background:**

Mechanical ventilation in the intensive care unit (ICU) is a life‐saving technique for patients with acute respiratory failure, but is also associated with a high incidence of complications in the injured lung. Currently, there is no widely used monitoring technique to guide the ventilator setting to facilitate a precision medicine approach or to provide a real‐time alert for developing adverse pulmonary conditions. Conventional ultrasound has been used as a thoracic bedside technology, but the lack of signal penetration into lung tissue results in images that often contain more information in their artifacts than in the images themselves. Perhaps the greatest obstacle to using traditional ultrasound in the ICU is the need for highly skilled technicians to perform the data collection. In contrast, low‐frequency ultrasound (50–500 kHz) has been shown to penetrate the lung, and can detect air trapping in patients with chronic obstructive pulmonary disease (COPD).

**Purpose:**

Here, we present a method of collecting low‐frequency ultrasound computed tomographic (USCT) data in vivo on a mechanically ventilated porcine model and computing tomographic reconstructions of airflow during tidal breathing and induced lung injuries. We evaluate the ability of the novel low‐frequency USCT system to image regional changes in sound speed in the thorax due to changes in airflow during tidal breathing and induced lung injuries. This represents the first study of low‐frequency tomographic ultrasound imaging in vivo and the first to produce tomographic images of ventilatory changes in vivo.

**Methods:**

USCT and computed tomography (CT) scan data were collected alternately on a mechanically ventilated Landrace pig weighing approximately 75 kg during tidal breathing, induced pneumothorax, atelectasis, and pleural effusion. The pneumothorax was induced by injecting air through a 5 mm thick intrathoracic tube inserted in the 8th posterior intercostal space. After removing the air, atelectasis was induced by ventilating the animal with a high concentration of oxygen and low tidal volumes. The pleural effusion was induced by injecting a saline solution through the tube. The USCT data were collected at 125 kHz using the USCT low‐frequency ultrasound tomography (LUFT) system on a transducer belt placed around the animal's thorax. Tomographic reconstructions were computed from the USCT data using a regularized refraction‐corrected Gauss‐Newton‐based time‐of‐flight reconstruction algorithm.

**Results:**

Cyclic changes in computed lung area during tidal breathing were demonstrated to agree with the respiratory rate on the mechanical ventilator. Reconstructed images computed at time steps during the procedure demonstrate regional changes consistent with what would be expected during the induced lung injury. No ground truth was available for images during the procedures since CT scans could only be taken before and after each established lung injury.

**Conclusions:**

In this work, we have demonstrated in the first in vivo study using a mechanically ventilated porcine animal model that low‐frequency ultrasound tomography has the ability to image regional changes in sound speed in the thorax corresponding to changes in airflow during tidal breathing and induced lung injury. The results show promise for using low‐frequency USCT as a bedside imaging technique in the future for patients with acute respiratory distress syndrome.

## 1 INTRODUCTION

Diagnostic imaging plays an essential role in detecting pathologies and monitoring lung disease evolution. Computerized tomography (CT) is the current gold standard for lung imaging and provides rich information about lung structure, and has been used to understand pathophysiological processes that underlie acute respiratory distress syndrome (ARDS), such as pulmonary edema,^1^ pleural effusion, pneumothorax, and atelectasis,^2^ but has several disadvantages including exposure of the patient to ionizing radiation, the need for evaluation of the images by a specialized technician, difficulties in transporting critically ill patients from the intensive care unit (ICU) to the radiology facility,^3^ and the lack of functional imaging capabilities. Chest x‐ray (CXR) can be an option as a bedside lung imaging tool, but it can only provide projection images, which can lead to misdiagnosis.^4^

An alternative to CT scans and CXR is ultrasound (US), which has been studied as a thoracic bedside monitoring technology for years.^5^, ^6^ However, US waves from conventional handheld scanners do not penetrate the lungs, and thus, the information contained in US images is mostly artifacts instead of the physiological structures themselves.^7^ Some artifacts have been found to carry information about potential pathologies, which spiked interest in exploring US as an option to monitor the progression of the disease and treatment of patients with the coronavirus disease of 2019 (COVID‐19).^8^, ^9^ For example, comet‐tail‐type artifacts arising from the pleural line (B‐lines) could be associated with interstitial lung disease^10^ and have been consistently reported in patients with COVID‐19 pneumonia, being an indication of its severity.^9^, ^11^ In addition, a sign of recovery could be the presence of A‐lines, a horizontal reverberation artifact that occurs beneath the pleural line and is associated with an aerated lung.^11^

All of the studies described above were performed using the conventional US imaging mode (B‐Mode), using reflection of sound waves in frequencies of 2 to 10 MHz. The technique produces a greyscale image, of which the pixel brightness is proportional to the amplitude of the logarithmically compressed envelope of echos produced by the interrogated tissues.^12^ The caveat of B‐Mode US is that it requires skilled technicians to perform the data collection and interpret the artifacts,^13^ which can be an obstacle to its use in the intensive care unit. In addition, the propagation of acoustic waves is more complex and produces much richer information than just their reflection.^14^, ^15^, ^16^ Tomographic techniques have the potential to interrogate the scattered field, which is a result of the interaction between the ultrasound pulse and the tissue through which it is propagating.^15^ Refraction, absorption, and attenuation are properties of acoustic waves through tissue, and sound speed and attenuation coefficients can be computed from the measured data.^14^

Tomographic ultrasound provides 2D images of the sound speed in the region of interest and is currently used clinically for breast cancer detection.^17^, ^18^, ^19^, ^20^ However, while acoustic waves in the MHz range are primarily reflected by the lung pleura, recent work has demonstrated that low‐frequency ultrasound (10–750 kHz) is transmitted through lung tissue and has promise for diagnostic use.^21^, ^22^ These properties motivate the application of tomographic low‐frequency ultrasound for pulmonary imaging. In this work, we present results from an animal study in which a novel low‐frequency ultrasound computerized tomography (USCT) system (LUFT^23^) that uses the Verasonics Vantage 64 low‐frequency system as the main hardware component. A belt of low‐frequency Tonpilz transducers is placed circumferentially around the region of interest, and signals are transmitted sequentially at the desired frequency. After signal processing, a time‐of‐flight (TOF) least‐squares tomographic reconstruction algorithm with refraction correction was applied to estimate the sound speed in the plane of the transducer array.

This work represents the first in vivo tomographic ultrasound images of ventilation and lung injury, as verified by a Google Scholar search with keywords low‐frequency ultrasound tomography in vivo. The most similar work is the paper *Whole‐Body Imaging Using Low‐Frequency Transmission Ultrasound*,^24^ in which in vivo tomographic images of four piglets acquired at 800 kHz are reconstructed in 3D. Images of the kidney, small intestine, and abdomen are presented, and the lung is visible in the abdominal images, but no lung pathologies were induced. In addition, in that work, the piglets were submerged in a water tank for data collection, while in our approach, a transducer belt was used. A further difference is that in the whole‐body imaging study, single snapshot images are computed to reconstruct anatomy, while we compute function images to show change. In particular, in this work, we present images showing sound speed changes due to an induced pneumothorax, pleural effusion, and atelectasis while the lung injury is occurring. This has never before been demonstrated with USCT. The images also represent the first low‐frequency tomographic images of physiological processes and the first use of low‐frequency Tonpilz transducers for in vivo functional imaging. It is the first demonstration that low‐frequency (LF) USCT can capture sound speed changes due to pathology as it develops, with data measured on a transducer belt, thus demonstrating its potential for bedside imaging.

## 2 METHODS

### 2.1 Hardware

**FIGURE 1:**
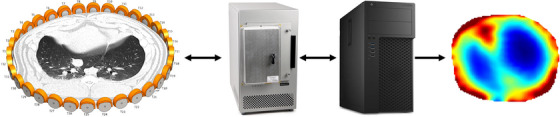
Simplified system block diagram of the low‐frequency USCT system illustrating belt placement around the thorax of the pig, the Verasonics system and host computer for the transmission and processing of the signals, and the resulting TOF reconstruction. TOF, time‐of‐flight; USCT, ultrasound computerized tomography.

The USCT LUFT system is described in ref. [23], where studies on tissue‐mimicking phantoms in the laboratory made of ballistic gelatin with high and low‐sound speed inclusions confirmed its ability to resolve objects several centimeters in diameter. The device uses a planar ring array of custom‐built low‐frequency transducers described in ref. [25] to transmit and receive sound waves at 125 and 156 kHz frequencies. A Verasonics Vantage 64 Low‐Frequency Research Ultrasound System generates the electric signals transmitted and received from the transducers. Figure 1 illustrates the main components of the system.

A belt with up to 32 transducers, equally spaced with a pitch distance of 37 mm, is attached to the subject. Each transducer is fired sequentially, while measurements are taken from all other transducers. The active transducer is driven by a parametric waveform with an amplitude of 80 V peak‐to‐peak, 40 half‐cycles in length, a center frequency of 125 kHz, and a duty cycle of 0.67. These parameters were chosen to optimize gain and signal‐to‐noise ratio (SNR) while preventing saturation of the analog‐to‐digital converter (ADC). The transmitting voltage and signal length were adjusted during the data collection session following a methodical approach. Initially, the transmitting voltage was set at a moderate level of 10 V peak‐to‐peak and 5 transmitted half‐cycles and was incrementally increased while monitoring the largest amplitude among the received signals. The goal was to achieve signal peaks reaching approximately 75% of the ADC's maximum value. This strategy ensured optimal utilization of the ADC's dynamic range, avoided signal saturation and clipping, and maintained the highest SNR achievable on that setup. Given the architecture of the Vantage 64 system's transmitting hardware and the transducers' underwater center frequency of 125 kHz, the bandwidth of 43 kHz (‐6dB), and beam divergence of 40 degrees,^25^ this configuration results in a transmitted waveform that closely approximates a sine wave and covers a desirable portion of the sample.

The wavefront travels through the phantom, reaching the passive transducers on the belt, generating electrical signals with an amplitude proportional to the sound pressure received at the transducer piston. The analog signals are then measured by the Vantage 64 system, filtered, digitized, and sent to a host computer running MATLAB. Data were acquired at a rate of 10.9 frames/s. The communication between the host computer and the hardware is bidirectional. The host controls all data collection parameters, such as the acquisition frequency, transmission pattern, and voltage, using a customized MATLAB script.

The raw digitized signals are then processed and prepared offline for reconstruction. First, the calibration coefficients, computed based on the transmitting and receiving transducers' underwater sensitivity, are applied to the raw signal using Equation (1).

(1) $${\text{Voltage}}_{c}=\frac{{\text{Voltage}}_{r}}{\frac{S_{R}}{S_{RMax}}.\frac{S_{T}}{S_{TMax}}},$$

where ${\text{Voltage}}_{c}$ is the corrected voltage, ${\text{Voltage}}_{r}$ is the measured signal, $S_{r}$ and $S_{t}$ are the receiving and transmitting sensitivities of the transducer pair, and $S_{RMax}$ and $S_{TMax}$ are the highest receiving and transmitting sensitivities in their respective categories. Next, the DC voltage and drifts are removed, and the signal is windowed and filtered using a 4th order Butterworth filter^26^ with a 175 kHz cutoff. The processed signal is then sent to a time‐of‐flight tomographic reconstruction algorithm, which computes the sound speed in each pixel of the phantom's domain. Finally, a 2‐D image is formed from the computed sound speeds.

To ensure safety, the mechanical and thermal indexes were computed and confirmed to be within FDA limits. The system operates optimally at frequencies of 125 kHz and 156 kHz, and with a driving voltage of 84.2 V peak‐to‐peak, it can generate a sound pressure of 0.438 MPa.^25^ The highest thermal index (TI) achieved at maximum power is 1.40,^27^ which represents 23% of the maximum value approved by the FDA.^28^

### 2.2 Vertebrate animal studies

A vertebrate animal study (Protocol number: 17‐7247A, Animal welfare assurance number: A3572‐01) was conducted at the Translational Medicine Institute, Colorado State University in Fort Collins, CO, USA, and aimed to analyze the behavior of the novel USCT system and the feasibility of the application of UCST for lung imaging and assessment of the development of respiratory complications. A Landrace pig weighing approximately 75 kg was obtained from a USDA‐approved vendor and used for the study. The Landrace species were chosen for their strong morphologic and hemodynamic behavior resembling human lungs. The study consisted of several steps of data collection with USCT and standard CT scan data to assess the pulmonary health of the subjects before and after induced acute lung injury.

#### 2.2.1 Preparation

Sedative agents were administered intramuscularly, and an intravenous catheter was placed in the auricular vein for the administration of intravenous anesthetics. Once anesthetized, the animal was orotracheally intubated using a laryngoscope and an endotracheal tube with an internal diameter of 7.5mm. Anesthesia was maintained with a continuous infusion of intravenous anesthetic and analgesic drugs and the animal was mechanically ventilated throughout the duration of anesthesia. Once the depth of anesthesia was considered adequate, a neuromuscular blocker was administered to avoid any interference, such as spontaneous breathing, during data collection. A board‐certified veterinary anesthesiologist continuously evaluated the anesthetic depth and quality via continuous monitoring of direct arterial blood pressure, heart rate and rhythm, arterial partial pressures of oxygen and $CO_{2}$, body temperature, muscle tone and movement; these vital signals and ventilator settings were annotated during the entire experiment. Next, the animal was placed in a CT scan bed, where the entire data study was conducted. The area between the 4th and 8th intercostal space was shaved and disinfected with alcohol to improve the acoustic coupling between the transducer and the animal's skin.

#### 2.2.2 Baseline assessment

USCT data were collected before any lung injury. The transducers and the animal's skin were coated with ultrasound gel, the belt was strapped to the pig's chest at mid‐lung height, and data were collected continuously during a 3‐min period at 125 kHz while the subject was mechanically ventilated at a rate of 20 breaths per minute and a tidal volume of 10 mL/kg. The arterial partial pressure of oxygen was measured to ensure adequate gas exchange. Next, the belt was removed, the ultrasound gel was cleaned from the skin, and self‐adhesive fiducial markers were carefully positioned where the transducers were previously located; a permanent marker was used to indicate the belt height, location, and the number of each transducer. After that, a CT scan was taken to evaluate the lung health condition of the animal before the acute lung injury was induced.

#### 2.2.3 Pneumothorax protocol

In a pneumothorax, air accumulates in the space between the lungs and the chest wall. To detect the presence of a pneumothorax, our study seeks to evaluate the system's ability to identify a region of slow sound speed (air) within the thoracic cavity that agrees with the location of the induced pneumothorax (confirmed through a CT scan). To accomplish that, minimally invasive surgery was performed to insert a 5 mm thick intrathoracic tube into the 8th posterior intercostal space. The transducer belt was prepared with ultrasonic gel and reapplied to the animal's thorax, and USCT data were collected continuously while the air was injected into the chest through the chest tube in increments of 60 mL until a change in systemic arterial pressure was detected. Once the pneumothorax was considered established, the data collection was stopped and restarted with a new dataset name. After that, the belt was removed, the fiducial markers were placed to represent the transducers, and a new CT scan was performed.

#### 2.2.4 Atelectasis protocol

Atelectasis was induced by ventilating the subject with a high concentration of oxygen (100% ${\mathrm{FiO}}_{2}$) and small tidal volumes (4 mL/kg). USCT data were collected during the entire procedure and restarted once the atelectasis was considered established by the veterinarian based on changes in the arterial partial pressure of oxygen (blood gas). After that, the belt was removed, the fiducial markers were placed, and a new CT scan was taken to confirm the health condition of the lungs.

#### 2.2.5 Pleural effusion protocol

In a pleural effusion, fluid accumulates in the space between the lungs and the chest wall. To detect the presence of this disorder, our study seeks to evaluate the system's ability to identify a region of high sound speed (fluid) within the thoracic cavity that agrees with the location of the induced pleural effusion (confirmed through a CT scan). USCT data were collected for approximately 14 min while injecting a saline solution into the thorax through the chest tube and restarted when a liquid volume of approximately 1.5 L was injected. Next, the belt was removed, the fiducial markers were installed, and a final CT scan was collected. After data collection was complete, following the American Veterinary Medical Association guidelines, an additional bolus of anesthetic was used preceding the injection of KCL to induce euthanasia through rapid and painless cardiac arrest.

### 2.3 Time of flight reconstructions

A refraction‐corrected Gauss‐Newton‐based time of flight reconstruction algorithm was chosen for its suitability for computing reconstructions of thousands of frames of data collected during breathing and induced lung injury. The open‐source method^29^ was used with generalized Tikhonov regularization consisting of the $L^{2}$‐norm of the Laplacian of the sound speed at the current iterate. We summarize the method here for completeness.

Let $T_{j}^{i}$ be the measured time of flight on transducer i when the jth transducer is firing. Let $\vec{T_{j}}={\left[T^{1},\text{…},T^{L}\right]}^{T}$ be the vector of measured time‐of‐flight data for L transducers. The travel time of the acoustic wave to the ith transducer when the jth transducer is firing, denoted by $\tau_{j}^{i}$, is modeled by the 2‐D eikonal equation. It is a first‐order nonlinear PDE satisfied by $\tau_{j}^{i}$

(2) $$|\nabla \tau_{j}^{i}\left(x,y\right)|=\frac{1}{c(x,y)},$$

where c(x,y) is the sound speed. Denoting the reciprocal of sound speed c(x,y) by s(x,y), also known as the “slowness”, we can write Equation (2) as

(3) $$\sqrt{{\left(\frac{\partial \tau_{j}^{i}}{\partial x}\right)}^{2}+{\left(\frac{\partial \tau_{j}^{i}}{\partial y}\right)}^{2}}=s\left(x,y\right)$$

for each pair of transducers {i,j}.

To estimate the sound speed throughout the region, consider the nonlinear least‐squares minimization problem

(4) $$\min_{\vec{s}}\left\{\sum_{k=1}^{L}{∥{\vec{\tau }}^{k}\left(\vec{s}\right)-{\vec{T}}^{k}∥}_{2}^{2}\right\},$$

where $\vec{s}\in R_{+}^{n}$ is the vector of pixel values of s, and the superscript of k represents the kth transmit (Tx) element. The gradient of (4) with respect to $\vec{s}$ is

$$2\sum_{k=1}^{L}J^{k}{\left(\vec{s}\right)}^{T}\left({\vec{\tau }}^{k}\left(\vec{s}\right)-{\vec{T}}^{k}\right),$$

where $J^{k}\left(\vec{s}\right)\in R_{+}^{L\times n}$ is the Jacobian matrix of $\vec{T}{\left(\vec{s}\right)}^{k}\in R_{+}^{L}$ for the kth firing pattern. That is, the (i,j)th entry of $J^{k}$ is given by

$$J^{k}\left(\vec{s}\right)\left(i,j\right)=\frac{\partial {\vec{T}}_{i}^{k}}{\partial s_{j}}.$$

Note that the entries of J represent the path length of each ray (row of J) over each pixel of $\vec{s}$ (column of J). Thus,

(5) $${\vec{\tau }}^{k}=J^{k}\left(\vec{s}\right)\vec{s}.$$

To compute the entries of $J^{k}$, the refracted ray paths between each source and receiver pair for slowness s(x,y) must be computed. A multistencils fast marching method (MSFM) proposed by Hassouna et al. in ref. [30] was used to solve the eikonal Equation (3) for a given s(x,y) and source location. Then, the spatial gradient of T(x,y) was used to trace the refracted ray path from the receiver back to the jth source, yielding $J^{k}\left(i,j\right)$. The MSFM computes the solution to (3) at each pixel by first defining stencils that cover each neighboring pixel and solving the eikonal equation along that stencil, and then selecting the solution that satisfies an upwind condition.

Substituting Equation (5) into Equation (4), we have

(6) $$\min_{\vec{s}}\left\{\sum_{k=1}^{L}{∥J^{k}\left(\vec{s}\right)\vec{s}-{\vec{T}}^{k}∥}_{2}^{2}\right\}.$$

Equation (6) can be solved using the iterative Gauss‐Newton method

(7) $${\vec{s}}^{i+1}=arg\left(\min_{{\vec{s}}^{i+1}}\right)\left\{\sum_{k=1}^{L}{∥J^{k}\left({\vec{s}}^{i}\right){\vec{s}}^{i+1}-{\vec{T}}^{k}∥}_{2}^{2}\right\},$$

where ${\vec{s}}^{i}$ is the approximation to the slowness from the ith iteration. However, solving this problem directly results in a nonsmooth and unstable reconstruction due to the ill‐posedness of the problem arising from the fact that the computed ray paths in $J^{k}$ do not include every pixel in the region.^29^ Thus, a regularization term is added to the objective function, and we solve

(8) $$\begin{array}{ccc} & & {\vec{s}}^{i+1}=arg\left(\min_{{\vec{s}}^{i+1}}\right)\\ & & \left\{\sum_{k=1}^{L}∥J^{k}\left({\vec{s}}^{i}\right){\vec{s}}^{i+1}-{\vec{T}}_{\text{obs}}{∥}_{2}^{2}+\alpha {∥R{\vec{s}}^{i+1}∥}_{2}^{2}\right\}. \end{array}$$

To promote smoothness of s(x,y), we chose a Laplacian penalty term, where R is chosen to be the discrete Laplacian matrix and α is a regularization parameter, chosen empirically. The problem is then formulated in stacked form and solved using the conjugate gradient method.

## 3 RESULTS

**FIGURE 2:**
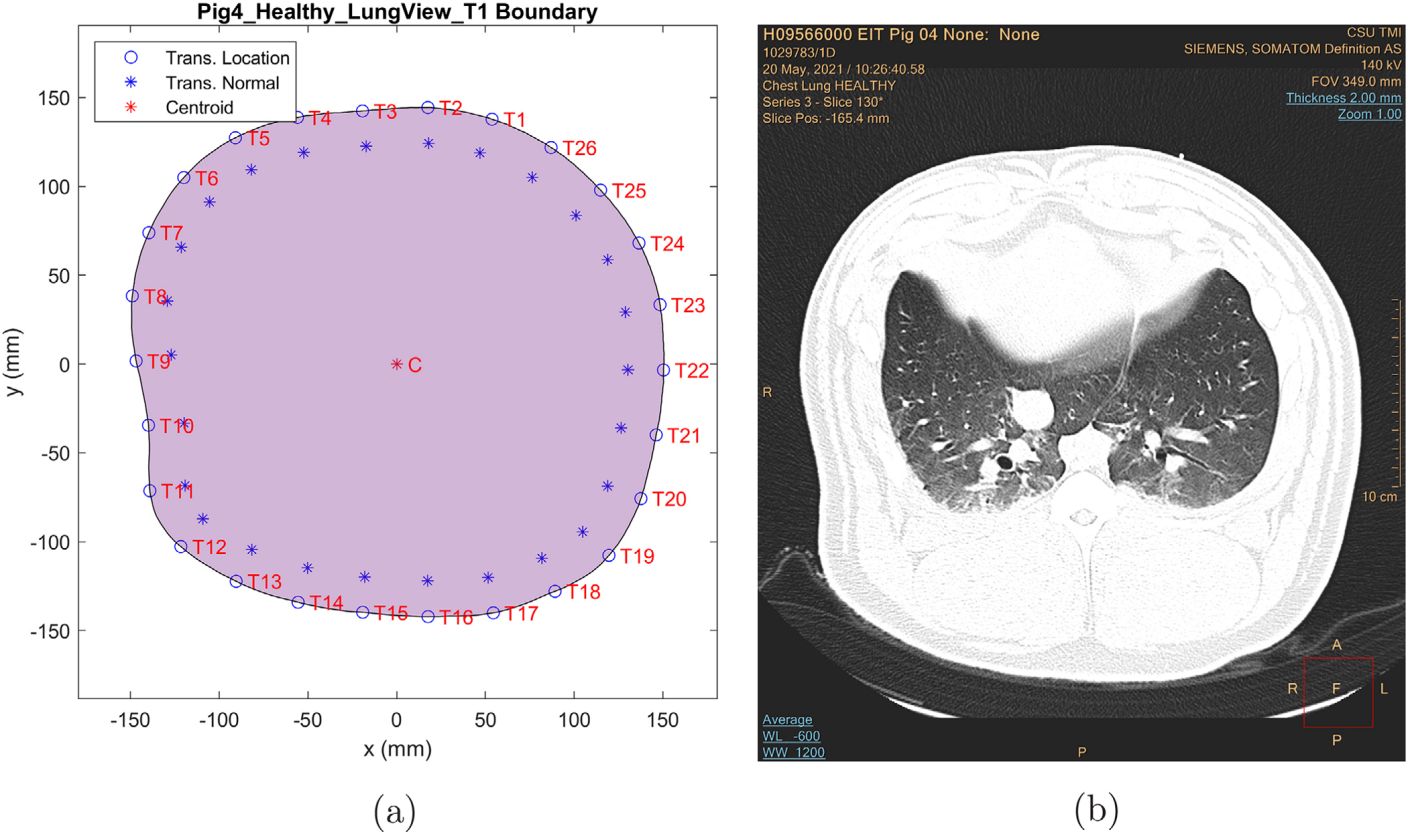
Boundaries and transducer numbering for data collection for baseline (a) and the CT scan used to compute the boundary (b). CT, computerized tomography.

**FIGURE 3:**
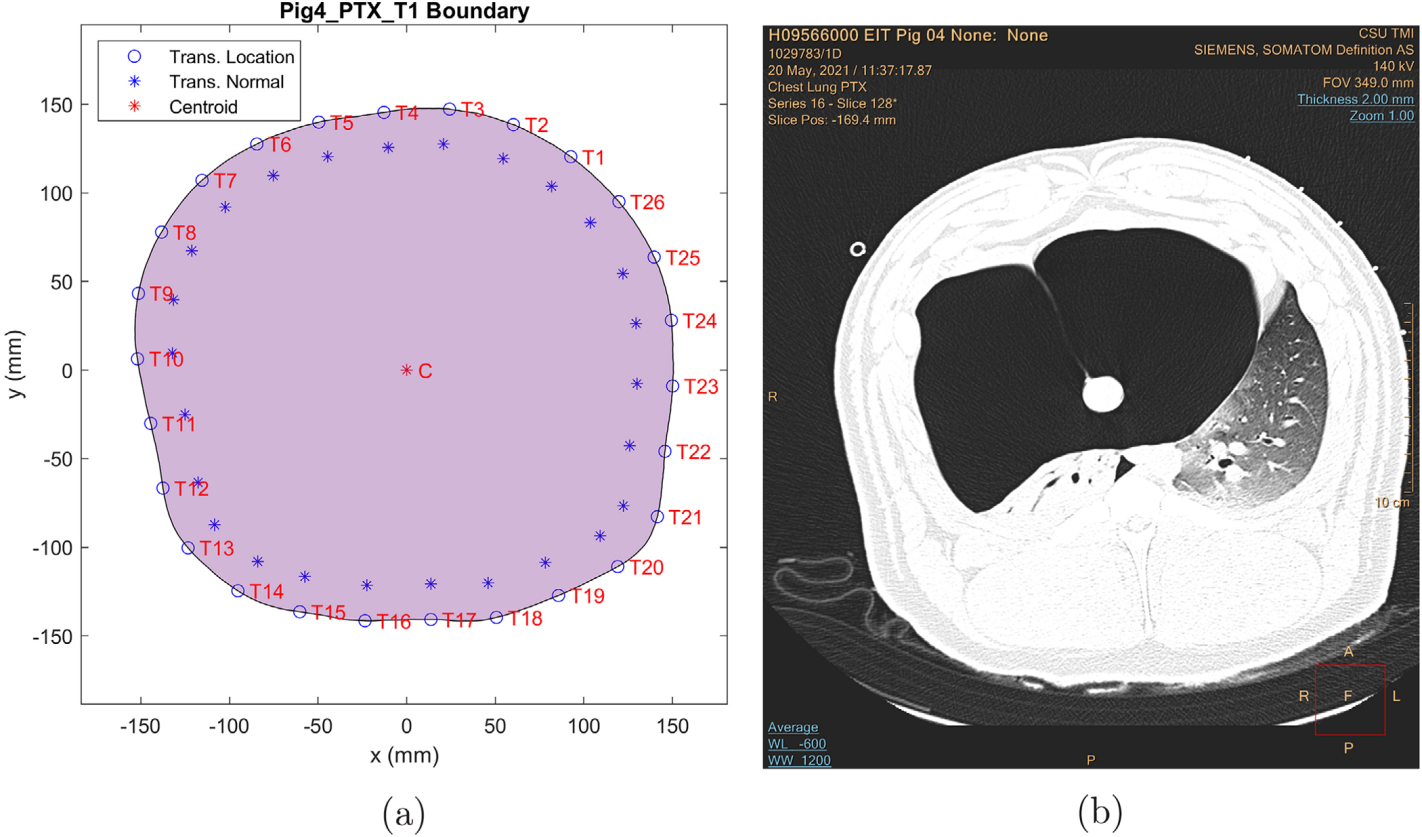
Boundaries and transducer numbering for data collection for induced pneumothorax (a) and the CT scan used to compute the boundary (b). CT, computerized tomography.

**FIGURE 4:**
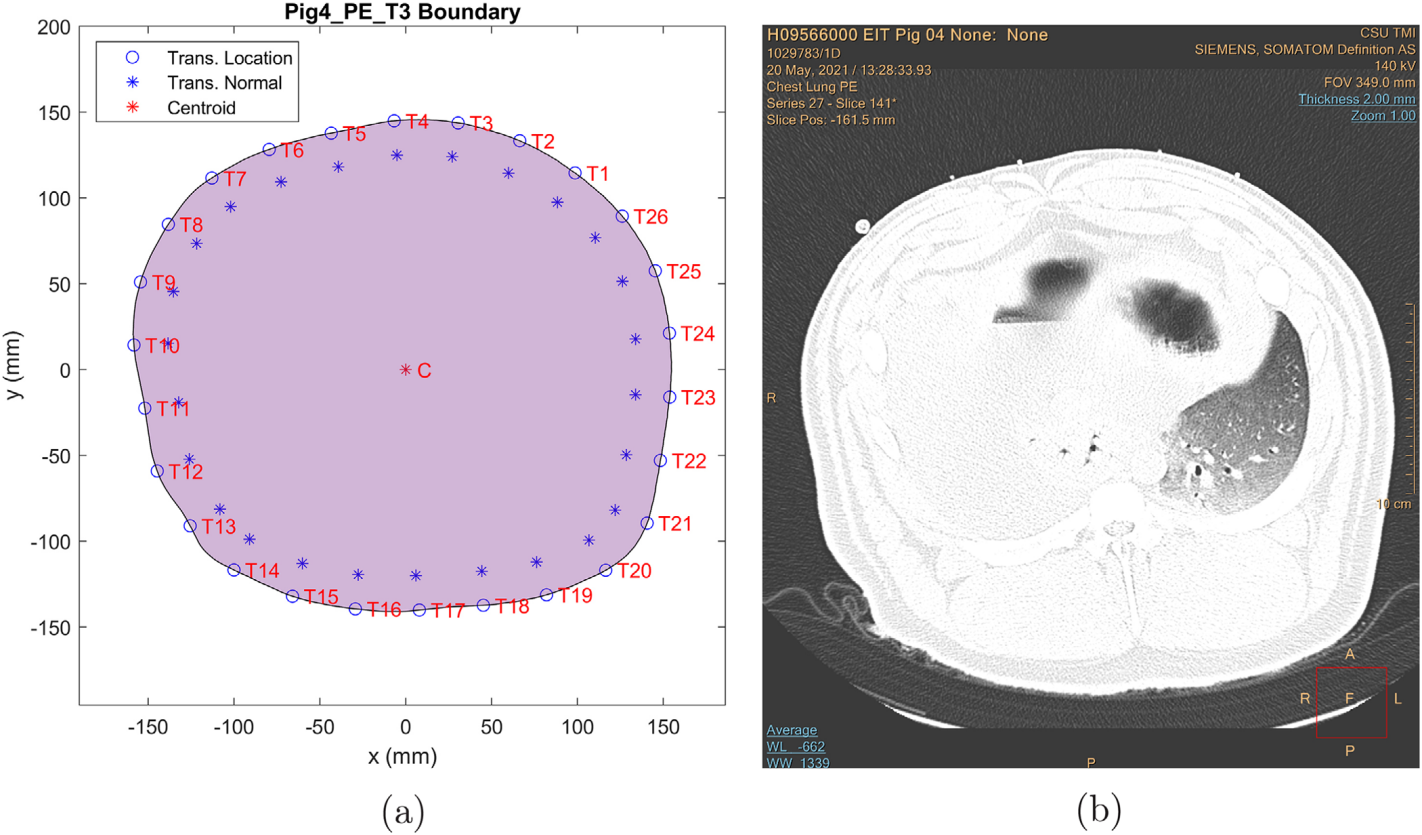
Boundaries and transducer numbering for data collection for pleural effusion (a) and the CT scan used to compute the boundary (b). CT, computerized tomography.

An approximation to the thorax boundary was computed for each belt placement using the CT scans and the locations of the fiducial markers in the scans. Each boundary is irregular and differs slightly from the others since the transducer belt needed to be removed before each CT scan and then re‐placed on the pig's thorax. Figures 2, 3, 4 show the calculated boundaries, which were used for the TOF reconstructions, and the CT scan used to compute it for the cases of baseline, pneumothorax (PTX), and pleural effusion (PE). Atelectasis is analogous and is omitted in the interest of brevity. All CT scan images and USCT reconstructions are displayed in digital imaging and communications in medicine (DICOM) orientation, in which the subject's left is on the reader's right. The boundaries were not the same for each case because the transducer belt was removed for the CT scan, and fiducial markers were placed at the location of the center of each transducer to avoid artifacts in the CT scan while recording the transducer location. The belt was replaced after each scan, but the locations of the transducers were not perfectly replicated with each replacement. The domain shape was created by choosing the CT scan slice containing the most fiducial markers and estimating the locations of the other transducers by analyzing the other slices. In the figures, the blue asterisks indicate the direction of the inward normal vectors, and the C marks the centroid of the domain. The CT scan slide used to construct each boundary is shown on the right.

### 3.1 Baseline

**FIGURE 5:**
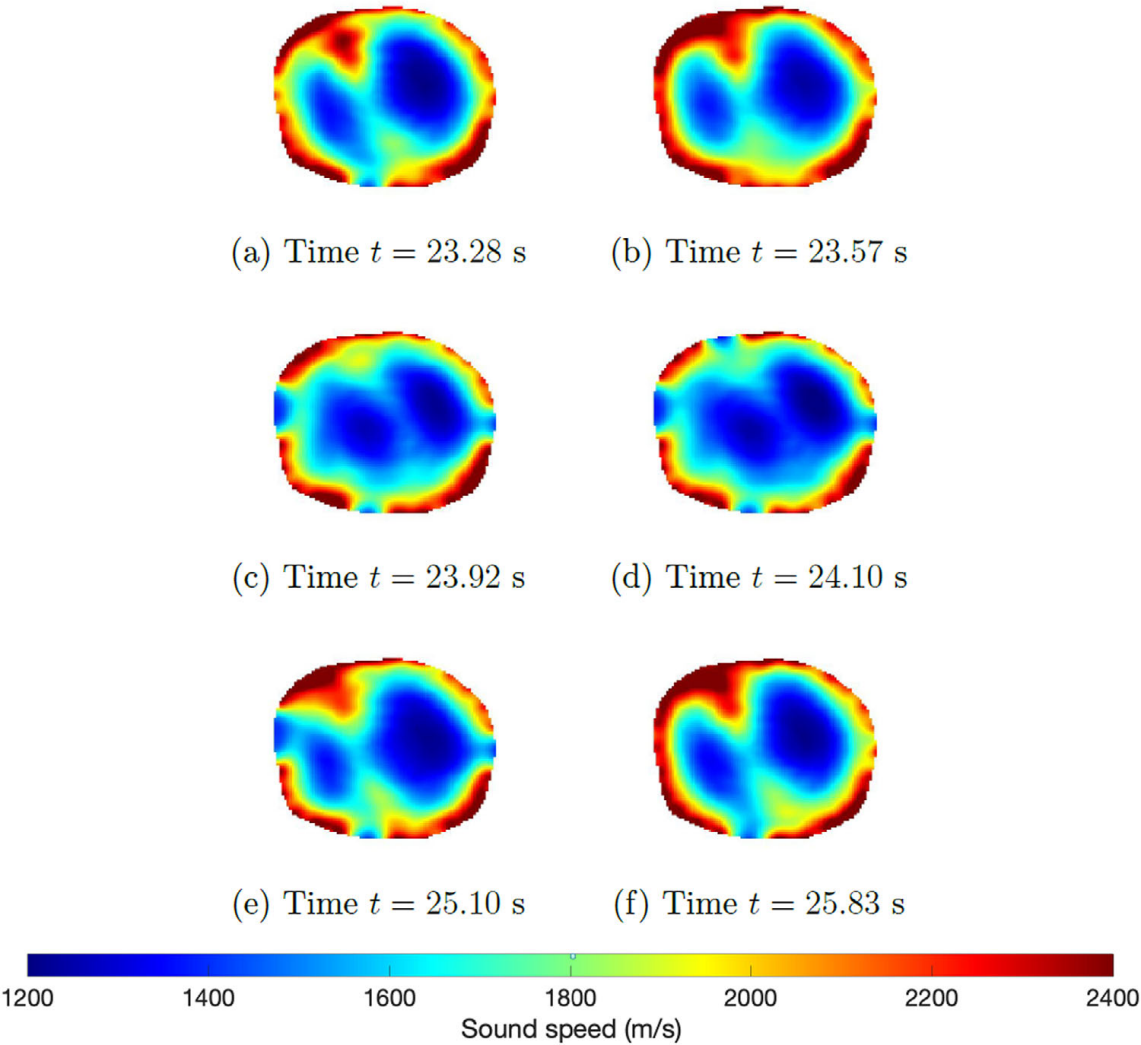
TOF sound speed reconstructions of the data collected during baseline; sequence of selected frames from one breathing cycle. TOF, time‐of‐flight.

**FIGURE 6:**
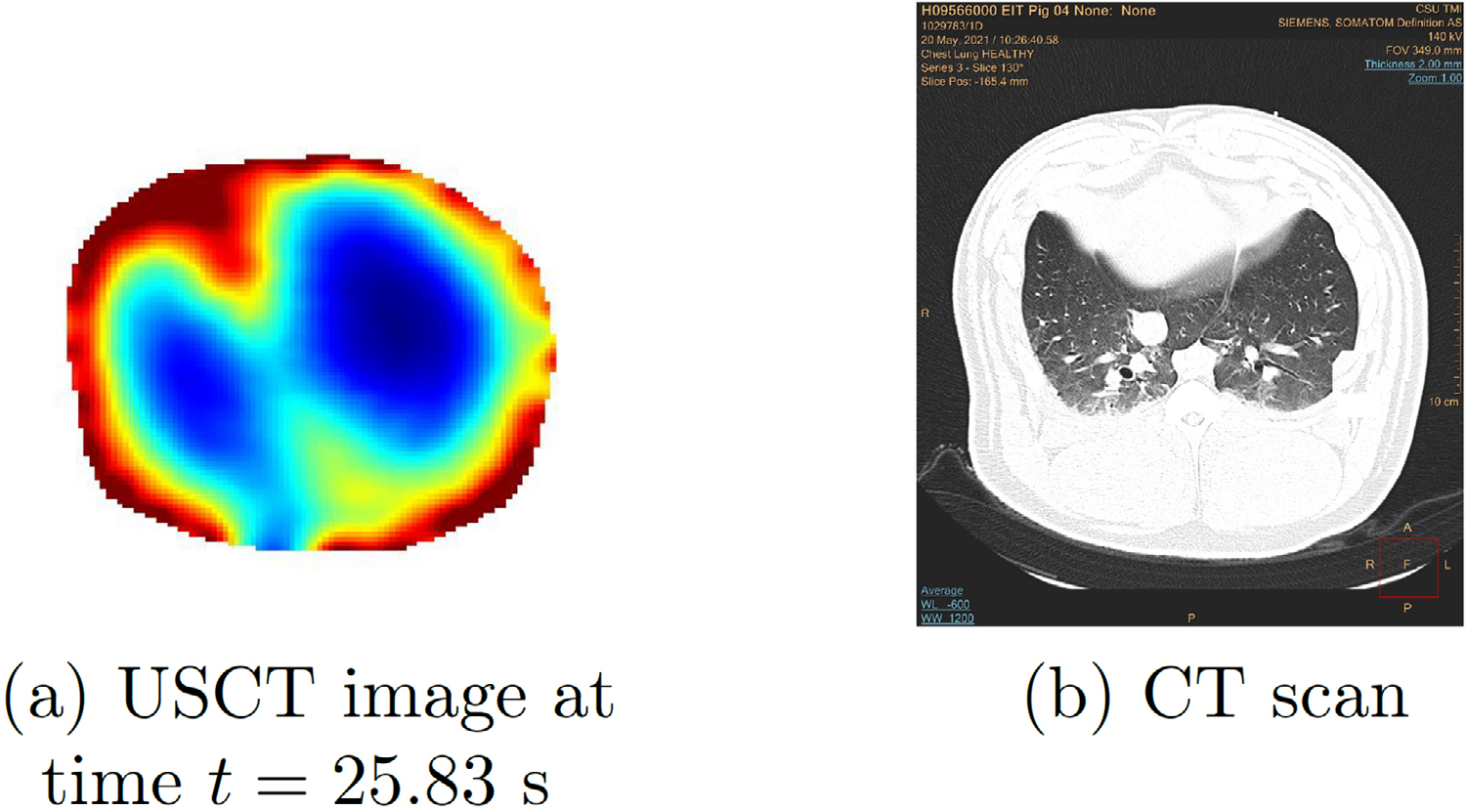
Left: USCT reconstruction during mechanical ventilation from Figure 5 on that same colorscale. Right: CT scan CT scan during mechanical ventilation at baseline. CT, computerized tomography; USCT, ultrasound computerized tomography.

A sequence of reconstructions of frames collected during mechanical ventilation prior to induced lung injury was reconstructed using TOF tomography. Six‐time snapshots of the TOF reconstructions are presented in Figure 5. Low sound speeds are plotted in blue, and high sound speeds are in red. The snapshots span one complete breathing cycle, ultimately returning to an image area very similar to that of the initial frame. A comparison to the CT scan taken to establish baseline before the induced lung injuries is found in Figure 6.

**FIGURE 7:**
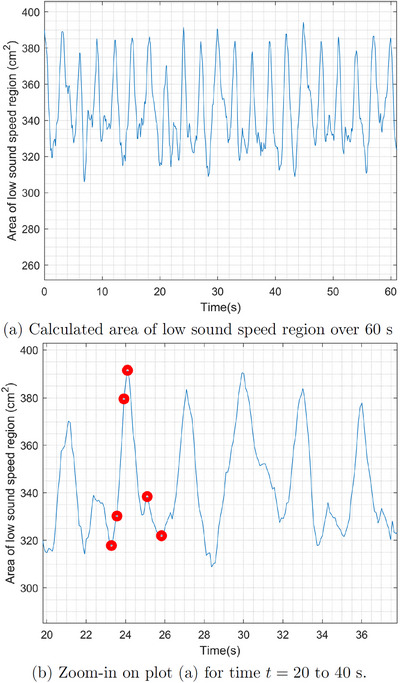
Low sound speed area computed from TOF sound speed reconstructions (DICOM) of the data collected during baseline. The red dots in (b) correspond to the time points for which the reconstructions are shown in Figure 5. DICOM, digital imaging and communications in medicine; TOF, time‐of‐flight.

A plot of the time trace of the lung area over 60 s of data collection is found in Figure 7. To estimate the lung area for each frame, first, an elliptical mask was superimposed to include only blue regions lying inside the red tissue region near the boundary, and then only pixels with sound speeds less than 1650 m/s were included in the calculation. The threshold value 1650 was chosen manually. Time traces of the plots of the areas were smoothed using a moving mean filter over 8 frames. Figure 7 zooms in on the plot of lung area from time t=20 s to t=40 s, with red dots indicating the times for which the images are plotted in Figure 5.

### 3.2 Pneumothorax

**FIGURE 8:**
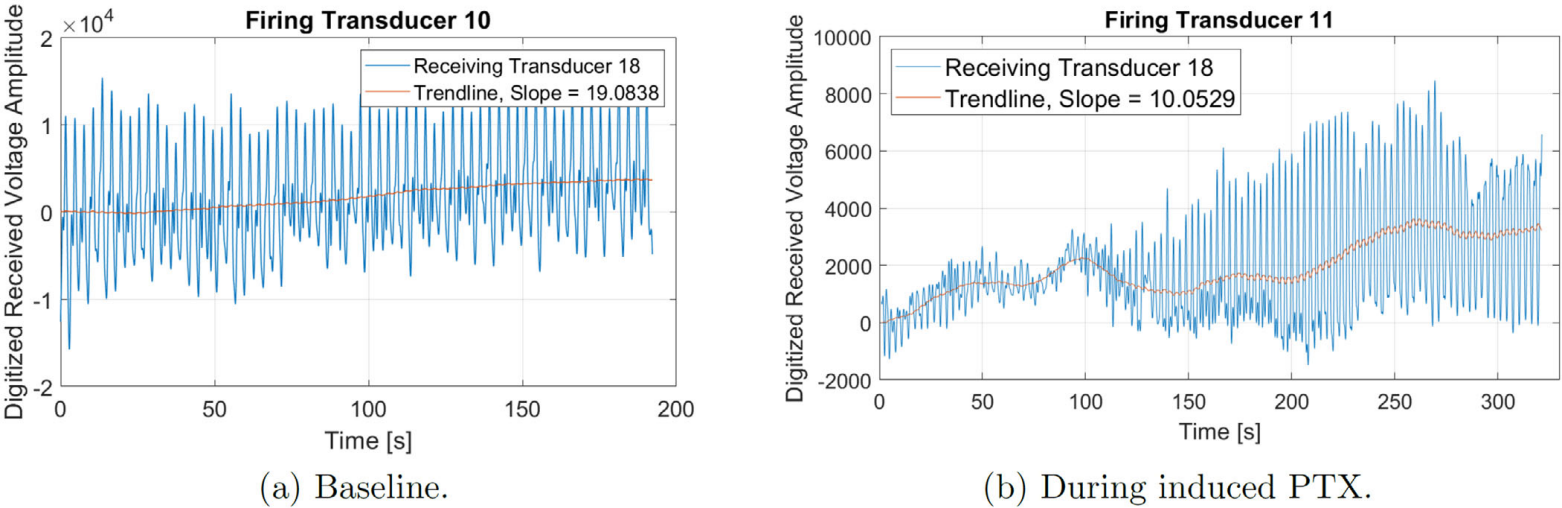
Amplitude of the received signal measured over time for different conditions: (a) during baseline tidal breathing prior to induced lung injuries and (b) during the induced pneumothorax. The trendline for each is plotted in red, and the slope of the line connecting the trendline at time t=0 and the final time plotted is found in the legend.

After the baseline data were collected, a pneumothorax was induced by injecting air into the chest in 60 mL increments over 5.4 min. USCT data were collected continuously for 5.4 min while the pneumothorax was induced. Figure 8b contains time traces of the computed peak‐to‐peak amplitude of the digitized voltage signal, subtracted by the mean value of the first two cycles on transducer 18 when transducer 11 is firing during the induction of pneumothorax. The time traces for the baseline case are plotted in Figure 8a.

**FIGURE 9:**
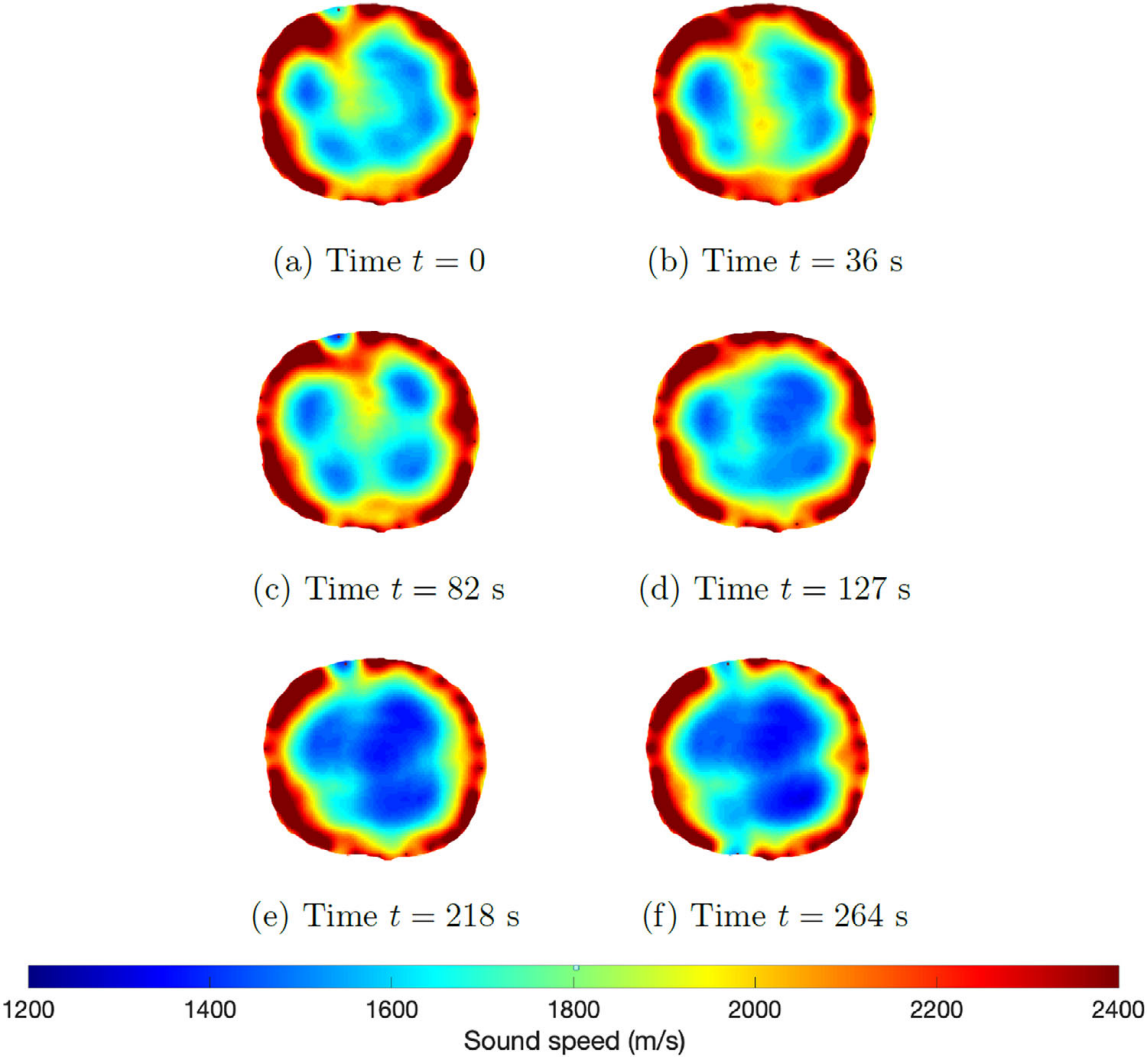
TOF sound speed reconstructions of the data collected during the pneumothorax protocol; sequence of selected frames showing the condition's progression. TOF, time‐of‐flight.

**FIGURE 10:**
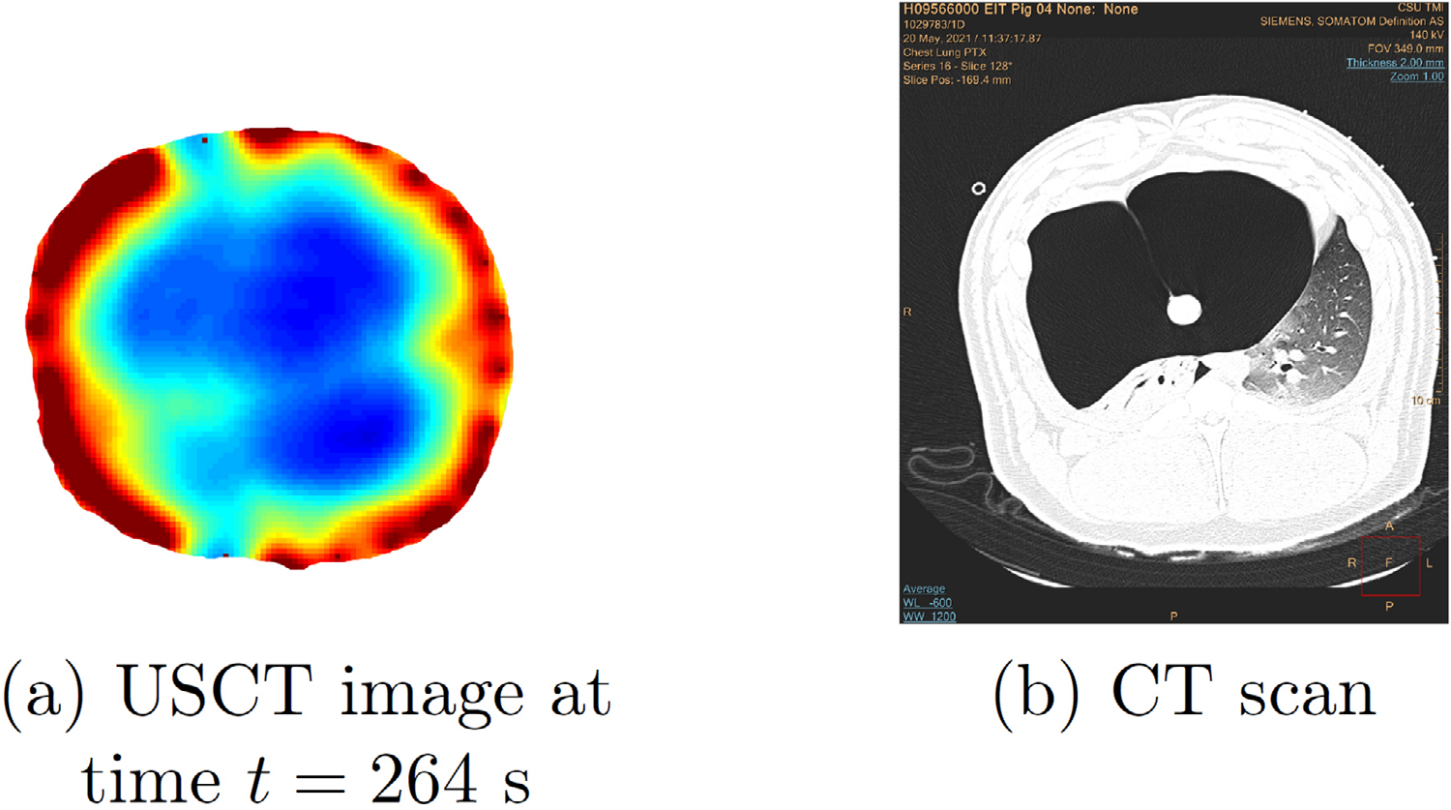
Left: USCT reconstruction at the final time after the pneumothorax was established from Figure 9 on that same colorscale. Right: CT scan after the pneumothorax was established. CT, computerized tomography; USCT, ultrasound computerized tomography.

**FIGURE 11:**
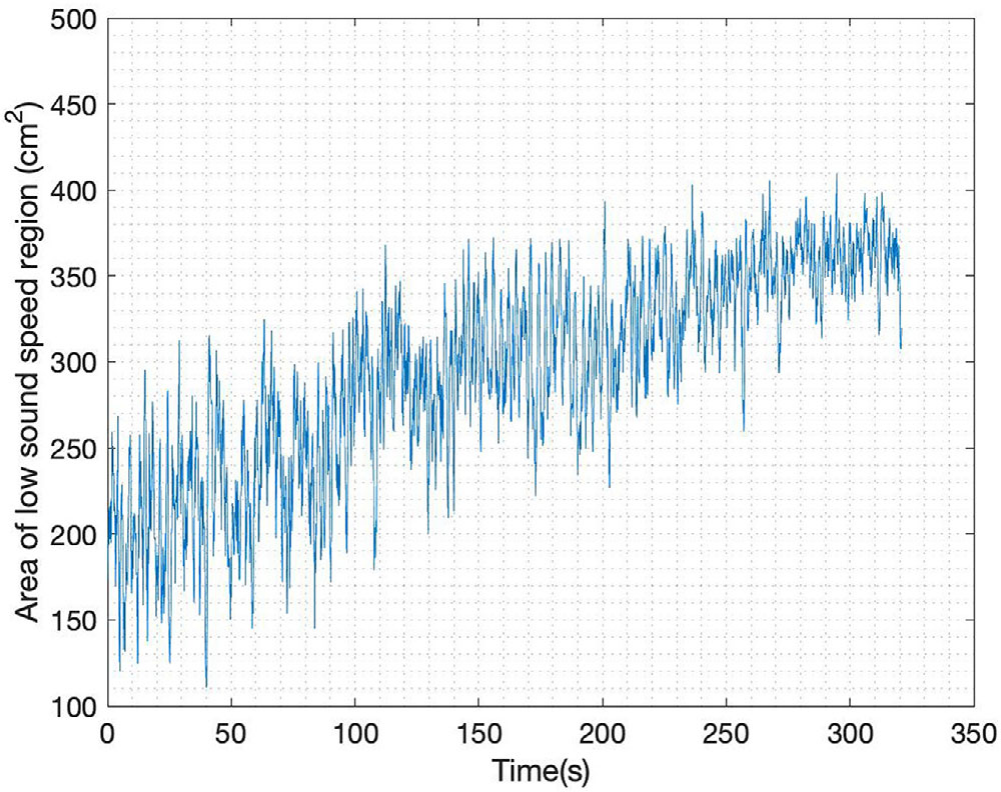
Calculated area of low sound speed region during the induced pneumothorax.

Six‐time snapshots of the TOF reconstructions are presented in Figure 9. Time t=0 is defined to be 1 min into the procedure when changes started to be observed, and so the final time t=264 s represents the final state, 5.4 min after the injection of air began. A comparison between the final USCT image and the CT scan taken after the pneumothorax was established is found in Figure 10. A plot of the time trace of the lung area over 321 s (about 5 min) of data collection is found in Figure 11.

### 3.3 Atelectasis

**FIGURE 12:**
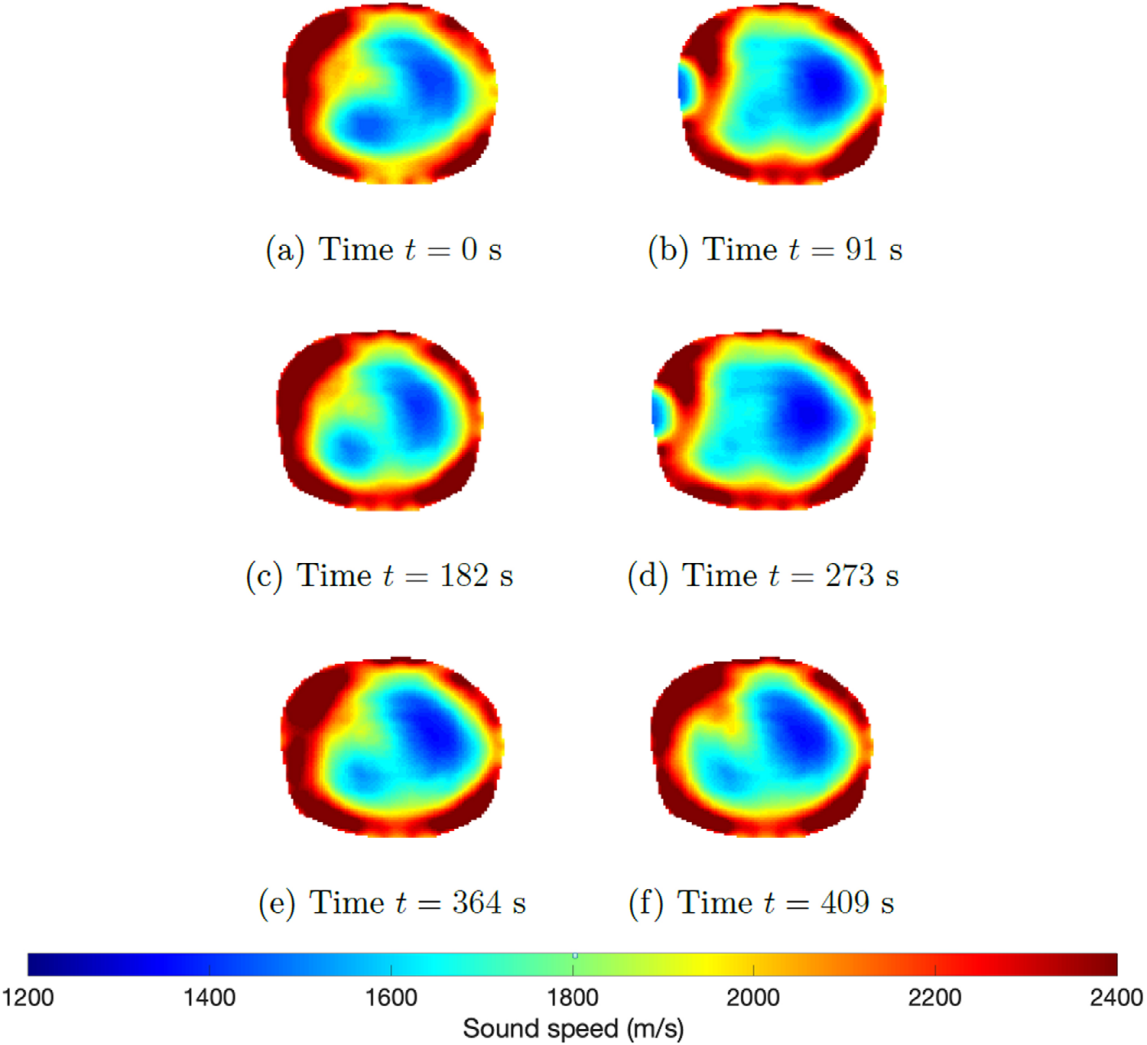
TOF sound speed reconstructions (DICOM) of the data collected during the atelectasis protocol; sequence of selected frames showing the condition's progression. DICOM, digital imaging and communications in medicine; TOF, time‐of‐flight.

**FIGURE 13:**
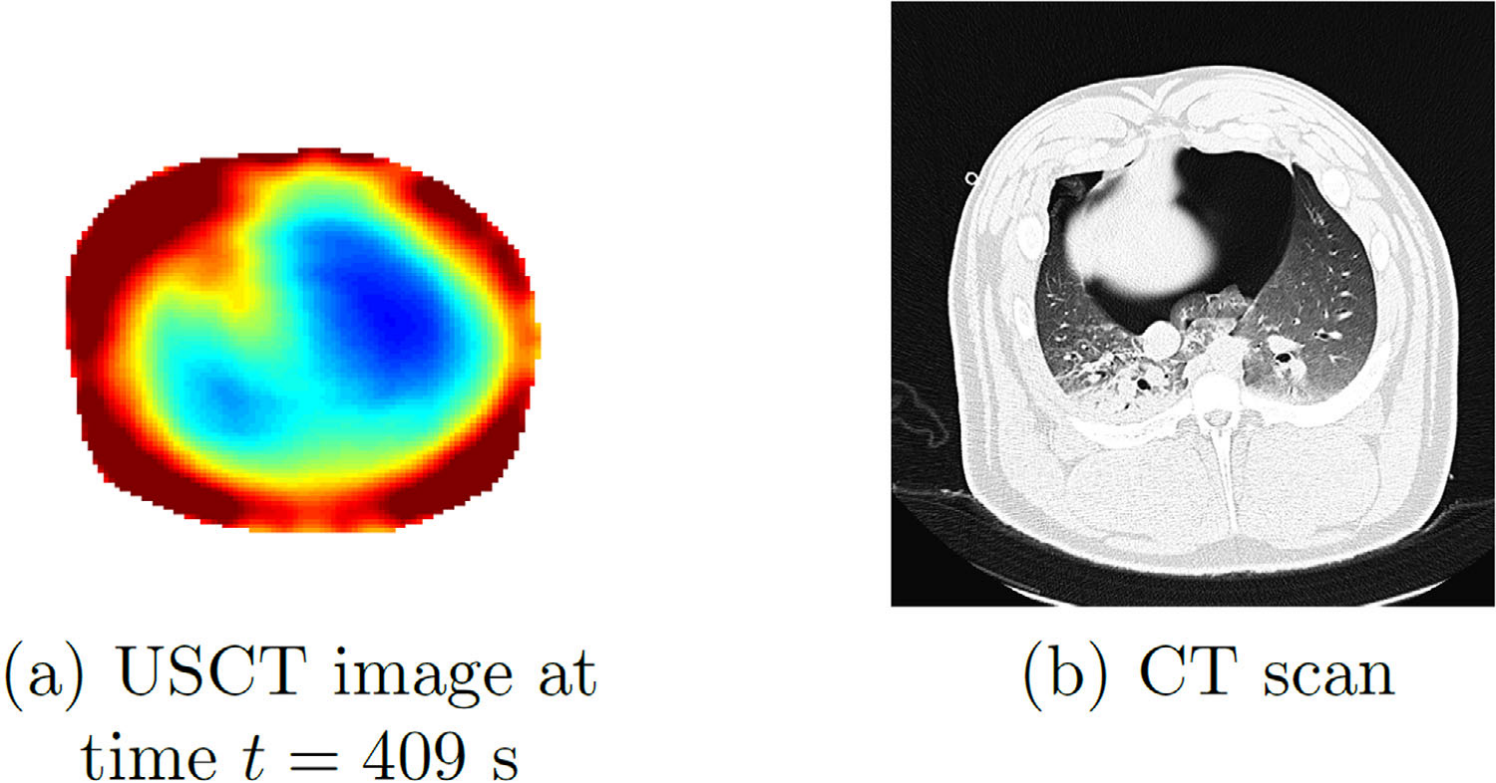
Left: USCT reconstruction at the final time after the atelectasis was established from Figure 12 on that same colorscale. Right: CT scan after the atelectasis was established. CT, computerized tomography; USCT, ultrasound computerized tomography.

**FIGURE 14:**
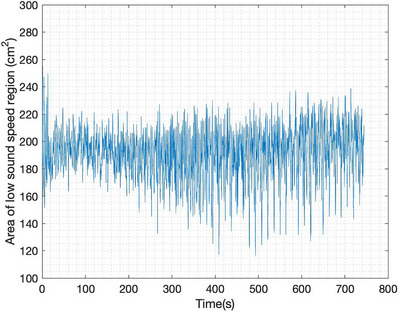
Calculated area of low sound speed region during the induced atelectasis.

Atelectasis was produced by ventilating the subject with a high concentration of oxygen (100% ${\mathrm{FiO}}_{2}$) and small tidal volumes (4 mL/kg). Six‐time snapshots of the TOF reconstructions from data collected during the induced atelectasis are found in Figure 12. In Figure 12, time t=0 corresponds to 5.5 min into the procedure, when changes were observed. The final time t=409 s is 6.8 min after the atelectasis has been established. The USCT image is plotted next to the CT scan in Figure 13 for comparison. A plot of the time trace of the lung area over 744 s (about 12 min) of data collection is found in Figure 14.

### 3.4 Pleural effusion

The pleural effusion was induced by injecting saline into the thorax through the chest tube over a period of approximately 14 min. Six‐time snapshots of the TOF reconstructions from data collected during the induced pleural effusion are presented in Figure 16. In Figure 16, time t=0 corresponds to 3.3 min into the procedure, when changes started to occur. The final time t=718 s is 12 min later when the pleural effusion has been established. The USCT image for the final time is plotted next to the CT scan in Figure 17 for comparison. A plot of the time trace of the lung area over 8716 s (about 145 min) of data collection is found in Figure 18.

**FIGURE 15:**
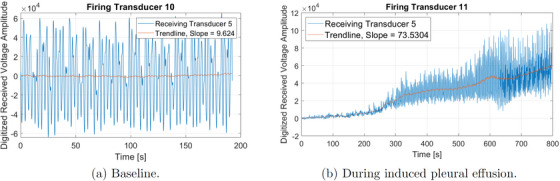
Amplitude of the received signal measured over time for different conditions: (a) during baseline tidal breathing prior to induced lung injuries and (b) during the induced pleural effusion. The trendline for each is plotted in red, and the slope of the line connecting the trendline at time t=0 and the final time plotted is found in the legend.

**FIGURE 16:**
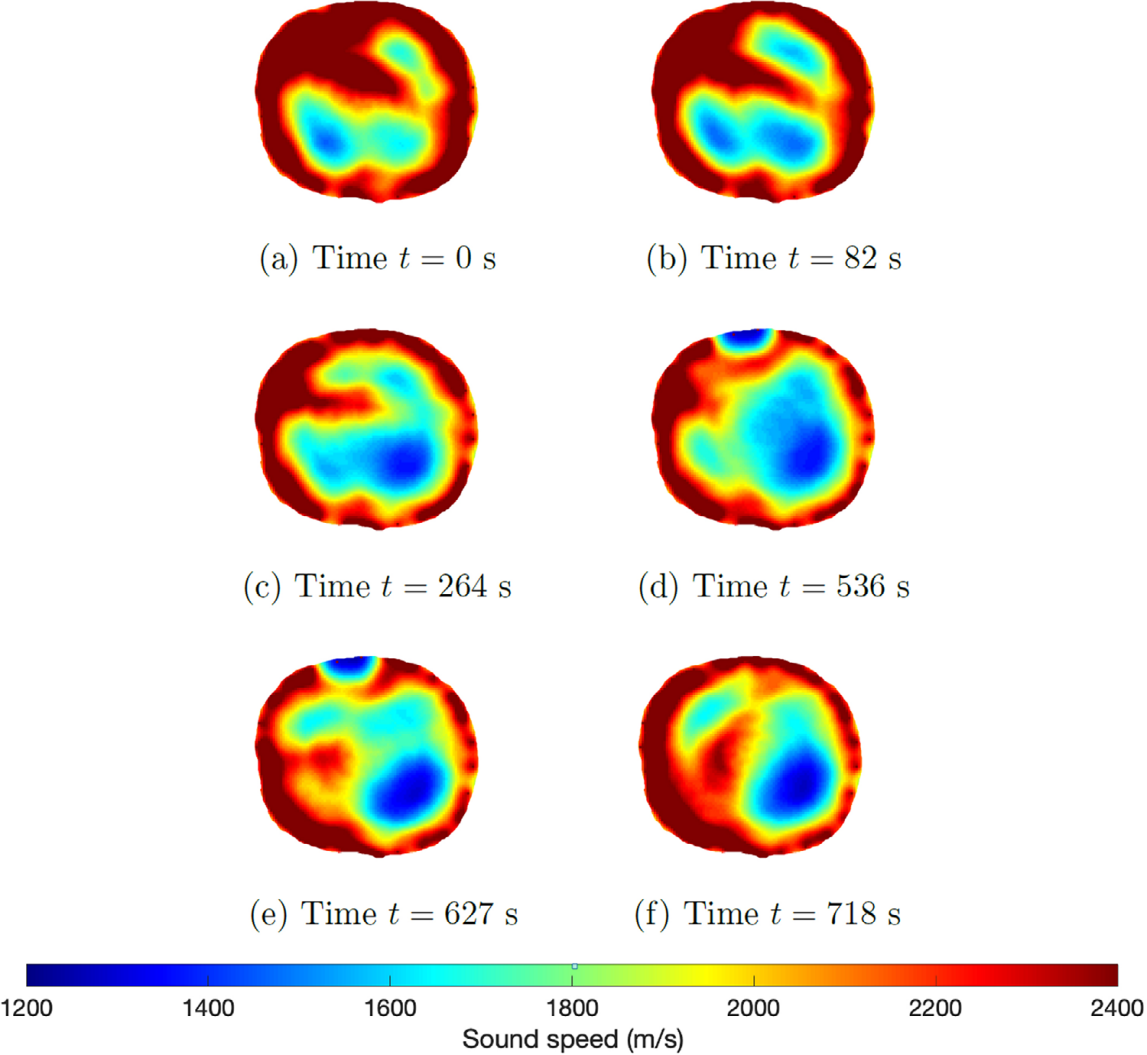
TOF sound speed reconstructions (DICOM) of the data collected during the pleural effusion protocol; sequence of selected frames showing the condition's progression. DICOM, digital imaging and communications in medicine; TOF, time‐of‐flight.

**FIGURE 17:**
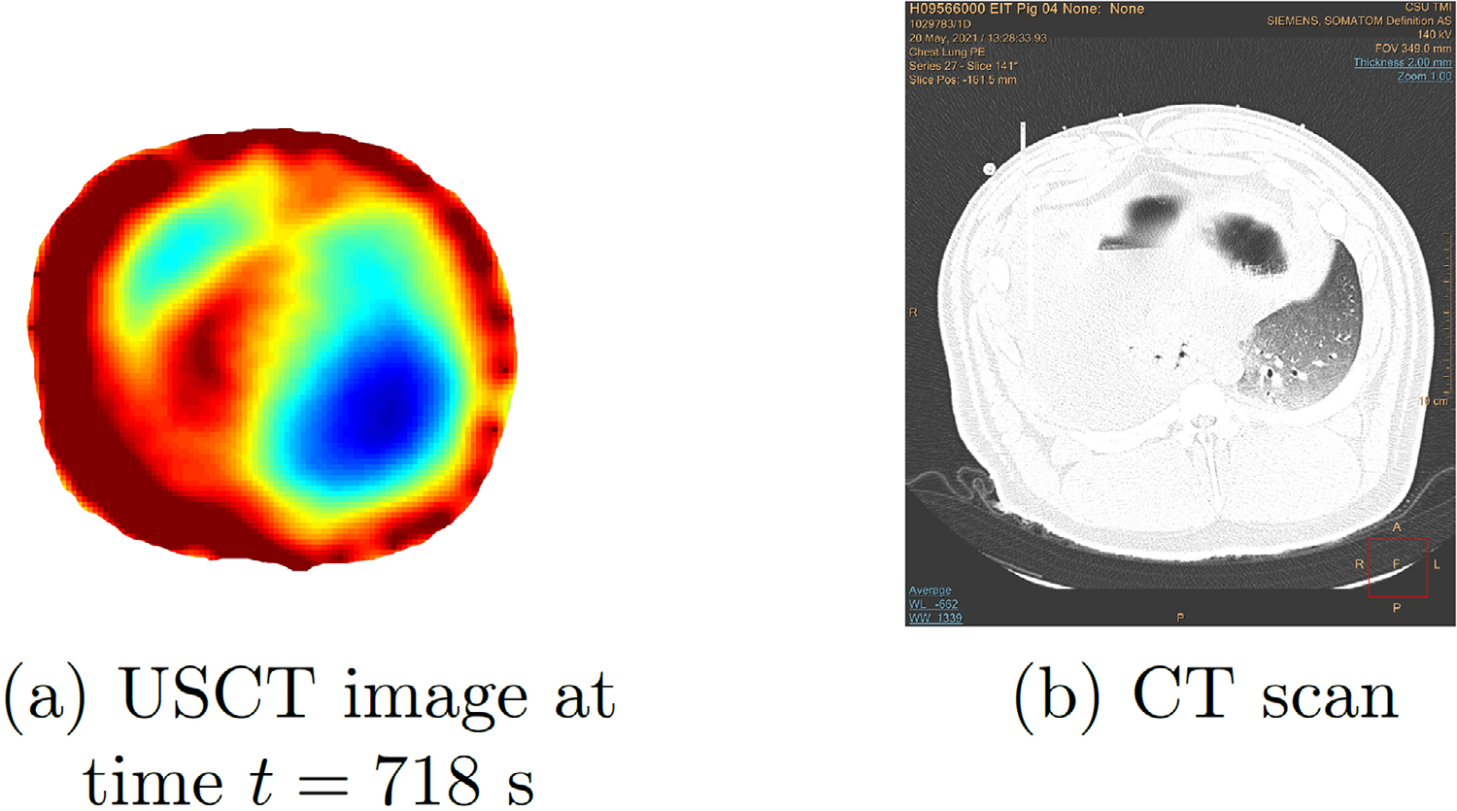
Left: USCT reconstruction at the final time after the pleural effusion was established from Figure 16 on that same colorscale. Right: CT scan after the pleural effusion was established. CT, computerized tomography; USCT, ultrasound computerized tomography.

**FIGURE 18:**
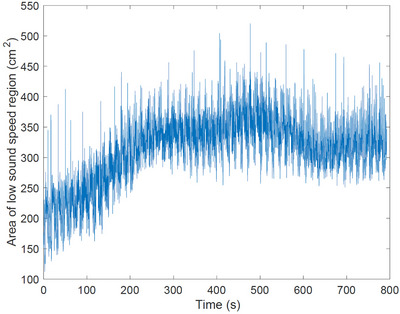
Calculated area of low sound speed region during the induced pleural effusion.

Figure 15b contains time traces of the computed peak‐to‐peak amplitude of the digitized voltage signal, subtracted by the mean value of its first two cycles, on transducer 5 arising when transducer 11 is firing while the pleural effusion is being induced. From Figure 4, one sees that transducer 11 is on the lower left side of the animal's body, and transducer 5 is on the upper left side of the front of the thorax. The time traces of the digitized voltage signals for the baseline case are plotted in Figure 15a.

## 4 DISCUSSION

The presence of low sound speed artifacts can be seen in some of the reconstructions of baseline, pneumothorax, and pleural effusion. We hypothesize that these could be due to motion artifacts that resulted in a change in transducer position, and this error in modeling resulted in a low estimate of the sound speed near that transducer. Also, in Figure 6, when comparing USCT with CT images, one observes a slight counterclockwise rotation of the USCT image relative to the CT scan. This is due to the fact that Transducer 1 (T1) is not located at 12:00 (that is, on the sternum) in any of the cases but rather at approximately 1:00 compared to the CT scan in each of the images. Since the reconstructions show T1 at 12:00, this results in a slight counterclockwise rotation relative to the CT scan.

It should also be noted that USCT does not offer the exact same field of view as CT. The CT slices are approximately 1 mm thick, and the USCT transducers are 1 cm in diameter. Also, the plane of the transducer belt is not perfectly aligned with the plane of the CT slice. The CT scans taken after the completion of each induced lung injury should also not be treated as ground truth because the condition of the animal's lungs is dynamic. After the lung injury was complete, and the last set of USCT data were collected, the transducer belt was removed prior to CT scanning. Removal of the belt involved moving and shifting the pig's position, which could have changed the distribution of air and fluid in the torso prior to CT scanning.

Potential sources of error that may impact the reconstructions include errors in the assumed transducer positions and domain shape and the absence of the first arrival times in some of the measured data. In particular, the higher applied voltages needed to attain a sufficient SNR on the pig seemed to result in a delay in the start of the recorded measurements on the transducers as part of the LF Verasonics Vantage system hardware. As a result, the signal corresponding to the first arrival time on the transducers neighboring the transmitting transducer is absent. We hypothesize this is the cause of the absence of the psoas major muscles (posterior muscles in the torso) in the reconstructed images.

The sound speed threshold 1650 m/s for the computation of the low sound speed area as plotted in Figures 7, 11, 14, and 18 was chosen manually and is just over half the maximum sound speed values in the images. Since the areas are computed to provide a means of quantifying the *cyclic changes* due to respiration and the *overall trends* in low sound speed area in the cases of lung injury, the exact choice of threshold to define the lung region is not critical. This threshold was used consistently, and constitutes a reasonable empirical choice, but somewhat lower or slightly higher results would yield the same cyclic behavior and overall trends.

### 4.1 Baseline

The six‐time snapshots of the TOF reconstructions in Figure 5 show subtle changes in aerated region during tidal breathing. The comparison to the CT scan in Figure 6 shows that the right lung is smaller than the left lung in both images. The plot of the time trace of the area of the low sound speed region over 60 s of data collection in Figure 7, provides an estimate of regional changes in the lung due to inhalation and exhalation. The frequency of oscillation confirms the presence of a cyclic pattern of lung volume, which was consistent with the ventilation rate of 20 breaths per minute and an inspiratory‐to‐expiratory ratio of 1:2, or an average breathing cycle of 3 s, with the inspiration and expiration phases lasting approximately 1 and 2 s, respectively. Given that the data were captured at a rate of 10.9 FPS, a complete breathing cycle should span approximately 33 frames, with 11 frames for inspiration and 22 for expiration.

### 4.2 Pneumothorax

From Figure 3 one sees that transducer 11 is on the right side of the thorax, and transducer 18 is on the left posterior region of the thorax. When comparing Figure 8a with Figure 8b, one of the clear differences is that there is an increase in the peak‐to‐peak amplitude (about 3.2 fold) and the DC level over time, suggesting that the injection of air into the animal's thorax was not only increasing the overall sound wave transmission, due to an increase in the DC level but also the amplitude of the breathing signal, due to the peak‐to‐peak amplitude increase. Those differences might have been caused by several factors, such as the compression of the animal's organs, which could improve the transmission by filling up gaps between them, the presence of the air bubble in the chest cavity which could increase the reflections by creating other tissue‐air interfaces or even the movement of the skin, due to chest inflation. When looking at the CT scan slice that represents the height where the belt was positioned, shown in Figure 10, a massive air bubble is visible in the animal's thorax causing the compression of the organs towards the pig's back. Therefore, since the path between transducers 11 and 18 was located across the region where the compression occurred, it could be responsible for the change in the acoustic wave transmission over time.

Upon analyzing the reconstructed frames between 199 and 3099 presented in Figure 9, the progression of the pneumothorax was evident. Over time, a consistent increase in the blue‐colored area was observed, likely due to the accumulation of air volume in the animal's thorax. This led to a collapse of the right lung, resulting in an overall decrease in sound speed in that area. Additionally, the high sound speed region near the 11:00 position, which was visible in the baseline reconstructions, can be seen in Figure 9a–c, but not in subsequent frames. This could be attributed to the compression of the tissue against the thoracic wall, which occurred due to the enlargement of the pneumothorax and was confirmed through the CT scan. A comparison between the final USCT image and the CT scan taken after the pneumothorax was established is found in Figure 10, and the results show good agreement between the large bolus of air in the anterior of the thorax and the blue region of the USCT image.

Additionally, a comparison between this reconstruction and the previous ones (baseline) reveals the absence of the high sound speed area observed at the 11:00 position in all baseline reconstructions. This may be attributed to the compression of the tissue that was originally present in that area. The relocation of tissues and lung collapse is confirmed through the CT scan, which resulted from the pressure exerted by the air injected during the pneumothorax protocol. The plot of the area of the low sound speed region in Figure 11 is much more irregular than that of the baseline plot in Figure 7. The area of the low sound speed region shows an overall increasing trend while the air is being injected, which is what one would expect to see during this procedure.

### 4.3 Atelectasis

Distinct changes in the aerated regions can be seen throughout the progression of the atelectasis in the six‐time snapshots of the TOF reconstructions from data collected during the induced atelectasis in Figure 12. Good agreement is seen between the CT scan and the final USCT image in Figure 13. In this case, it is seen in Figure 14 that the area of the low sound speed region stays fairly constant on average. This may be expected since no bolus of air or fluid is added to the lung during the induced atelectasis; changes were made to the ventilator by increasing the amount of oxygen delivered and decreasing the tidal volumes.

### 4.4 Pleural effusion

From Figure 4, one sees that transducer 11 is on the lower left side of the animal's body, and transducer 5 is on the upper left side of the front of the thorax. When looking at the time traces of the digitized voltage signals for the baseline case plotted in Figure 15a, an increase in the peak‐to‐peak voltage and DC over time is clear. However, in this case, the difference was in the order of 35‐fold instead of 3.2. The slope of the trendline was also much higher, reaching a value of 73.5 instead of 10.05. The reason for such an increase in transmission could be explained by the presence of a large amount of fluid on the pathway between the transmitting (T11) and receiving (T5) transducers, which can be seen in the CT scan in Figure 17. The attenuation coefficient of water can be up to 450 times smaller than certain tissues.^31^ Thus, that could be a reasonable explanation for the transmission improvement in this experiment.

Upon analyzing the time snapshots from time t=0 to t=718 s, clear evidence of the pleural effusion progression was observed. A consistent migration of the high sound speed region, initially concentrated around the 10:00 position, can be seen and is likely due to the spread of the fluid injected in the chest tube, which was inserted in the right side of the pig's thorax. At time t=0, it appears that most of the fluid was concentrated in the top right region of the thorax, while the subsequent frames indicate fluid migration towards the back and center of the thoracic cavity. The final frame showed fluid accumulation around the left lung and the thoracic cavity center, which agrees with the CT scan shown in Figure 17. Since these are 2D cross‐sections of the three‐dimensional lung, a linear increase in sound speed cannot be expected since the saline can flow throughout the lung and out of the plane of the transducers.

The post‐procedure CT scan reveals the presence of fluid throughout the thoracic cavity, except the left lung and the area immediately above it (between 10:00 and 2:00). Similarly, the TOF reconstruction demonstrated a low sound speed region at the 4:00 position, corresponding to the aerated lung position on the CT scan. Additionally, the high sound speed area located in the center of the reconstruction coincided with the fluid region identified by the CT scan, while the low sound speed area in the northwest region corresponded with the fluid‐free region (CT).

One sees from Figure 18 that the low sound speed region increases initially, but then shows some overall oscillation in the overall trend. We hypothesize that the initial increase in the area of the low sound speed region is because the fluid need not accumulate in the plane of the transducers; it will be subject to gravity and air will be pushed around in the lung as more fluid is added, resulting in a nonlinear trend and some overall oscillation. This irregularity in air distribution is also supported by the images in Figure 16 during the induced pleural effusion.

## 5 CONCLUSIONS

In this work, we have demonstrated in a mechanically ventilated porcine animal model that the low‐frequency ultrasound tomography system LUFT has the ability to image regional changes in sound speed in the thorax corresponding to changes in airflow during tidal breathing and induced lung injury. Cyclic changes in computed lung area during tidal breathing were demonstrated to agree with the respiratory rate on the mechanical ventilator. Reconstructed images showed regional changes in aeration during the induced lung injury. No ground truth was available for images during the procedures since CT scans could only be taken before and after each established lung injury, and changes in air distribution could have occurred between the time of USCT data collection and CT scanning. Potential sources of error in the reconstructions include errors in the assumed transducer positions, domain shape, and absence of first arrival times on some of the signals. Future work will address these issues.

## CONFLICTS OF INTEREST STATEMENT

The authors have no conflicts to disclose.

## Data Availability

The datasets used during the current study are available from the corresponding author on reasonable request
